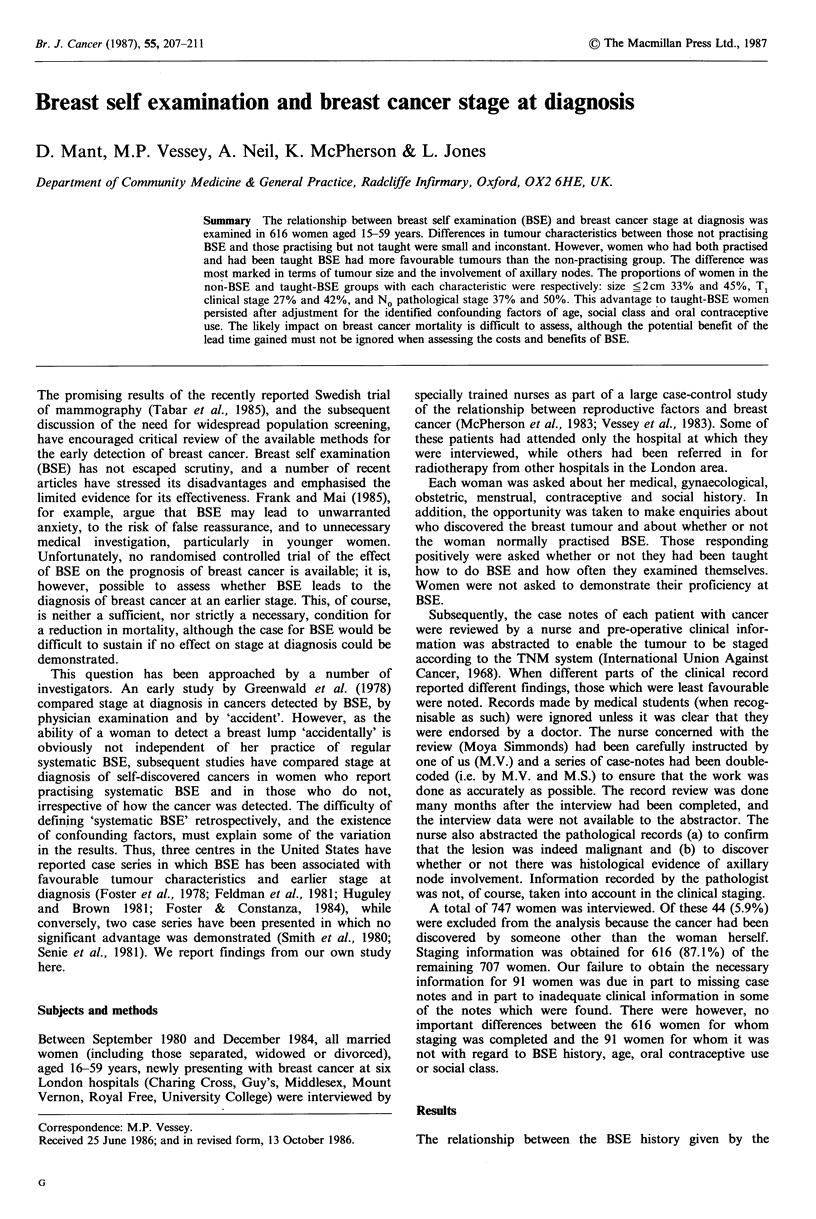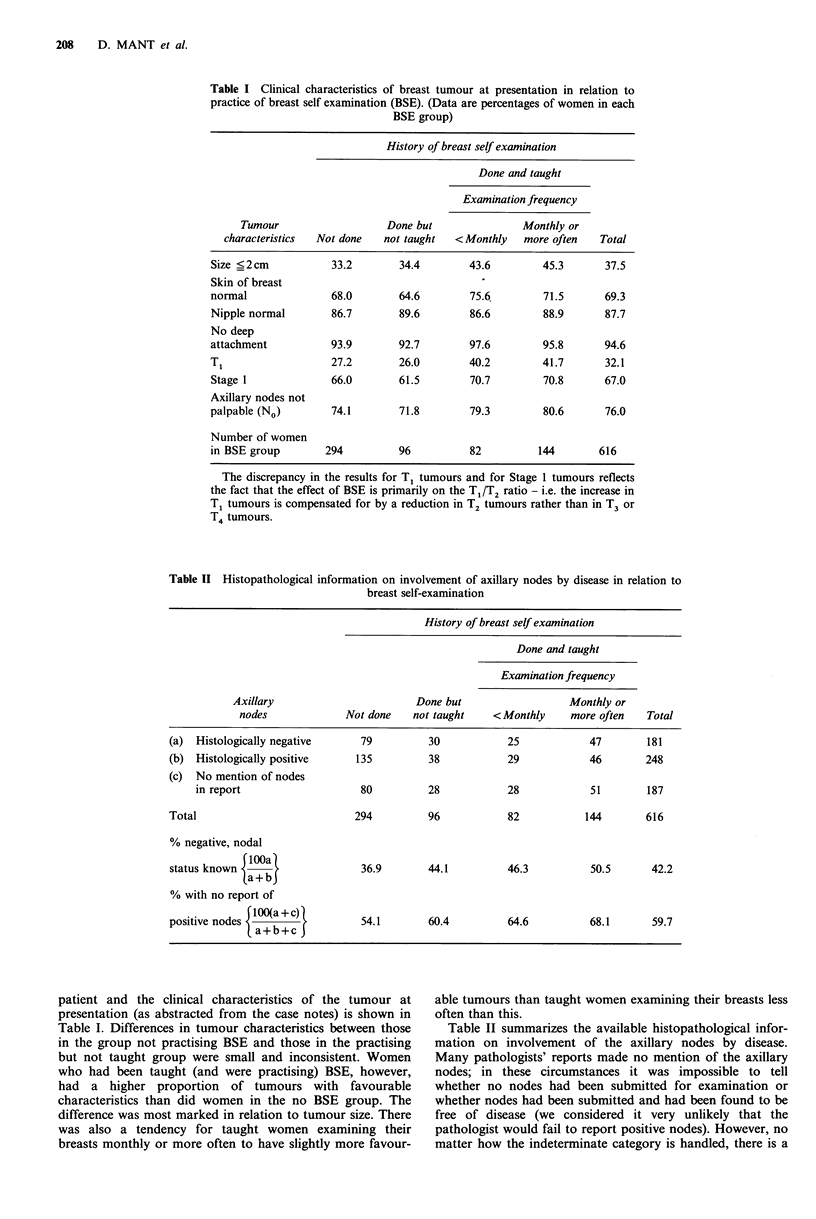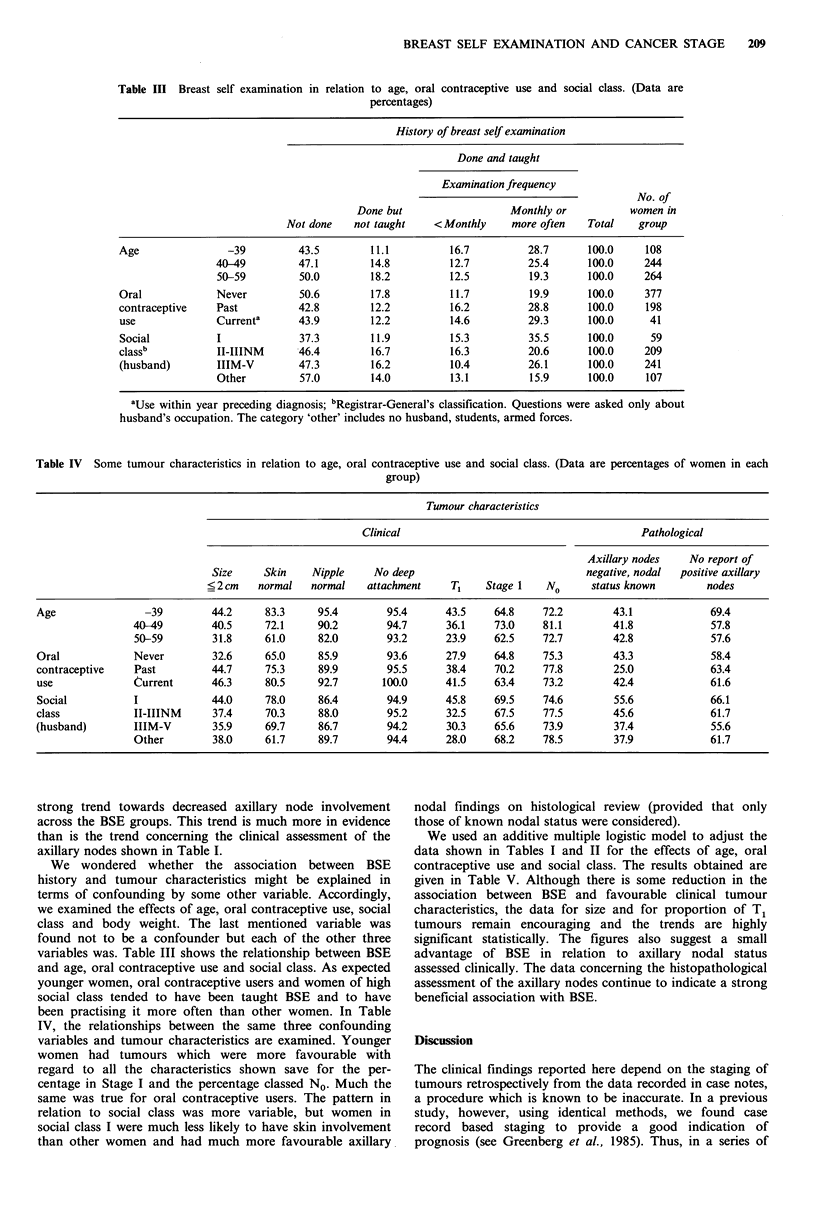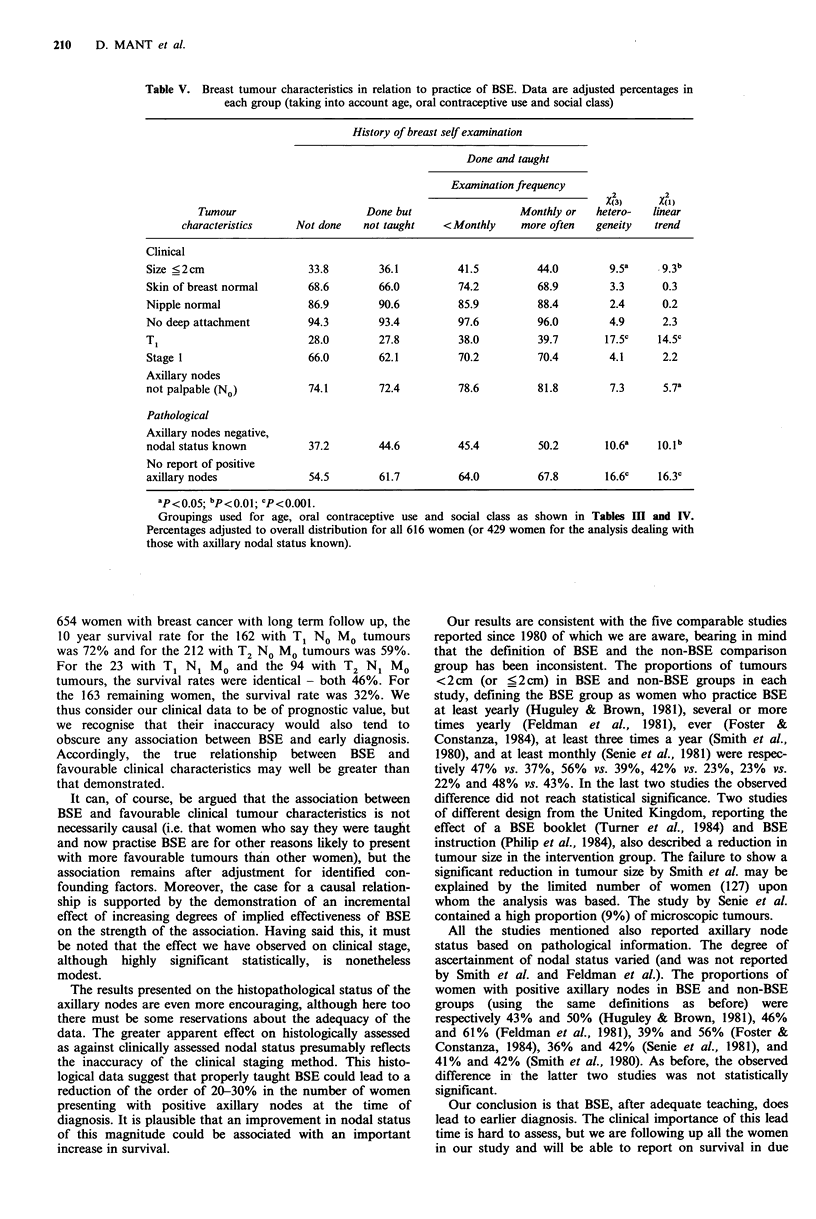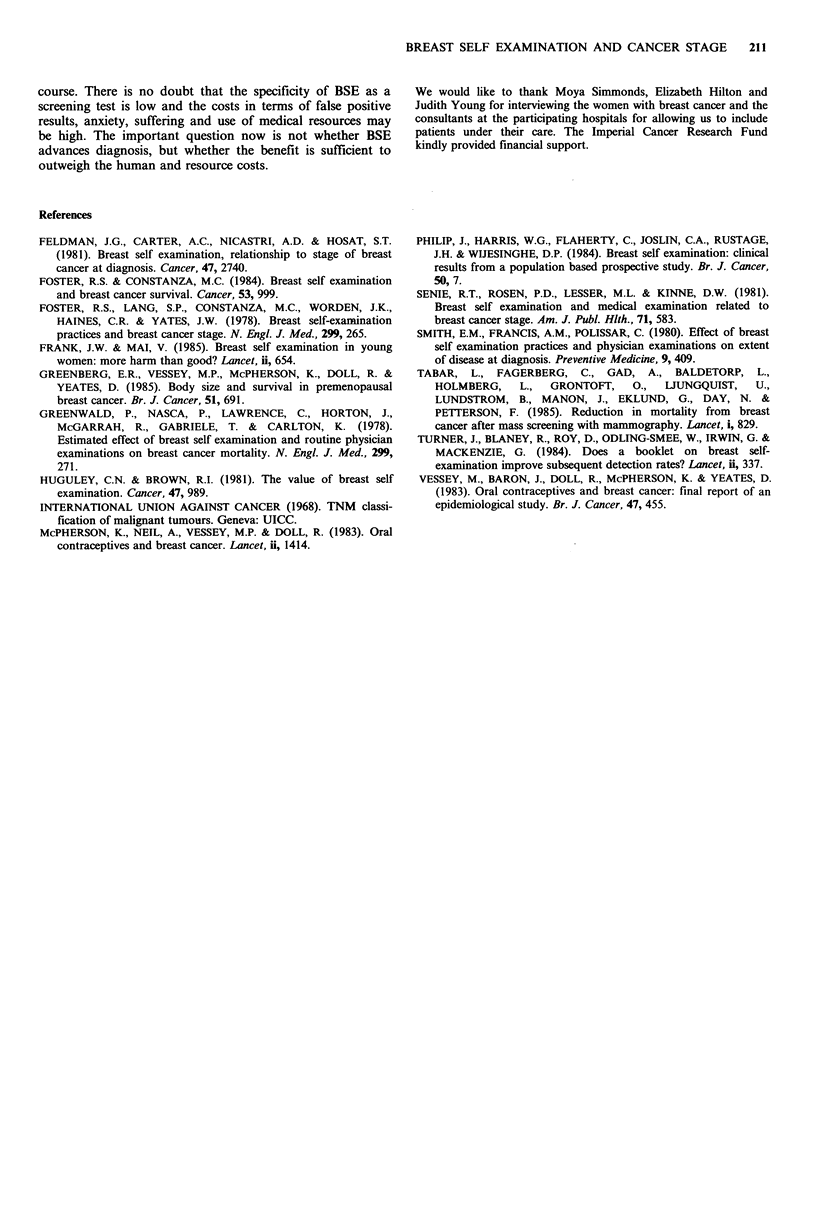# Breast self examination and breast cancer stage at diagnosis.

**DOI:** 10.1038/bjc.1987.39

**Published:** 1987-02

**Authors:** D. Mant, M. P. Vessey, A. Neil, K. McPherson, L. Jones

## Abstract

The relationship between breast self examination (BSE) and breast cancer stage at diagnosis was examined in 616 women aged 15-59 years. Differences in tumour characteristics between those not practising BSE and those practising but not taught were small and inconstant. However, women who had both practised and had been taught BSE had more favourable tumours than the non-practising group. The difference was most marked in terms of tumour size and the involvement of axillary nodes. The proportions of women in the non-BSE and taught-BSE groups with each characteristic were respectively: size less than or equal to 2 cm 33% and 45%, T1 clinical stage 27% and 42%, and N0 pathological stage 37% and 50%. This advantage to taught-BSE women persisted after adjustment for the identified confounding factors of age, social class and oral contraceptive use. The likely impact on breast cancer mortality is difficult to assess, although the potential benefit of the lead time gained must not be ignored when assessing the costs and benefits of BSE.


Br. J. Cancer (1987), 55, 207–211 © The Macmillan Press Ltd., 1987

### Breast self examination and breast cancer stage at diagnosis

D. Mant, M.P. Vessey, A. Neil, K. McPherson & L. Jones

*Department of Community Medicine & General Practice, Radcliffe Infirmary, Oxford, OX2 6HE, UK.*

**Summary** The relationship between breast self examination (BSE) and breast cancer stage at diagnosis was examined in 616 women aged 15–59 years. Differences in tumour characteristics between those not practising BSE and those practising but not taught were small and inconstant. However, women who had both practised and had been taught BSE had more favourable tumours than the non-practising group. The difference was most marked in terms of tumour size and the involvement of axillary nodes. The proportions of women in the non-BSE and taught-BSE groups with each characteristic were respectively: size $\leq 2$ cm 33% and 45%, $T_1$ clinical stage 27% and 42%, and $N_0$ pathological stage 37% and 50%. This advantage to taught-BSE women persisted after adjustment for the identified confounding factors of age, social class and oral contraceptive use. The likely impact on breast cancer mortality is difficult to assess, although the potential benefit of the lead time gained must not be ignored when assessing the costs and benefits of BSE.

The promising results of the recently reported Swedish trial of mammography (Tabar *et al.*, 1985), and the subsequent discussion of the need for widespread population screening, have encouraged critical review of the available methods for the early detection of breast cancer. Breast self examination (BSE) has not escaped scrutiny, and a number of recent articles have stressed its disadvantages and emphasised the limited evidence for its effectiveness. Frank and Mai (1985), for example, argue that BSE may lead to unwarranted anxiety, to the risk of false reassurance, and to unnecessary medical investigation, particularly in younger women. Unfortunately, no randomised controlled trial of the effect of BSE on the prognosis of breast cancer is available; it is, however, possible to assess whether BSE leads to the diagnosis of breast cancer at an earlier stage. This, of course, is neither a sufficient, nor strictly a necessary, condition for a reduction in mortality, although the case for BSE would be difficult to sustain if no effect on stage at diagnosis could be demonstrated.

This question has been approached by a number of investigators. An early study by Greenwald *et al.* (1978) compared stage at diagnosis in cancers detected by BSE, by physician examination and by 'accident'. However, as the ability of a woman to detect a breast lump 'accidentally' is obviously not independent of her practice of regular systematic BSE, subsequent studies have compared stage at diagnosis of self-discovered cancers in women who report practising systematic BSE and in those who do not, irrespective of how the cancer was detected. The difficulty of defining 'systematic BSE' retrospectively, and the existence of confounding factors, must explain some of the variation in the results. Thus, three centres in the United States have reported case series in which BSE has been associated with favourable tumour characteristics and earlier stage at diagnosis (Foster *et al.*, 1978; Feldman *et al.*, 1981; Huguley and Brown 1981; Foster & Constanza, 1984), while conversely, two case series have been presented in which no significant advantage was demonstrated (Smith *et al.*, 1980; Senie *et al.*, 1981). We report findings from our own study here.

### Subjects and methods

Between September 1980 and December 1984, all married women (including those separated, widowed or divorced), aged 16–59 years, newly presenting with breast cancer at six London hospitals (Charing Cross, Guy's, Middlesex, Mount Vernon, Royal Free, University College) were interviewed by specially trained nurses as part of a large case-control study of the relationship between reproductive factors and breast cancer (McPherson *et al.*, 1983; Vessey *et al.*, 1983). Some of these patients had attended only the hospital at which they were interviewed, while others had been referred in for radiotherapy from other hospitals in the London area.

Each woman was asked about her medical, gynaecological, obstetric, menstrual, contraceptive and social history. In addition, the opportunity was taken to make enquiries about who discovered the breast tumour and about whether or not the woman normally practised BSE. Those responding positively were asked whether or not they had been taught how to do BSE and how often they examined themselves. Women were not asked to demonstrate their proficiency at BSE.

Subsequently, the case notes of each patient with cancer were reviewed by a nurse and pre-operative clinical information was abstracted to enable the tumour to be staged according to the TNM system (International Union Against Cancer, 1968). When different parts of the clinical record reported different findings, those which were least favourable were noted. Records made by medical students (when recognisable as such) were ignored unless it was clear that they were endorsed by a doctor. The nurse concerned with the review (Moya Simmonds) had been carefully instructed by one of us (M.V.) and a series of case-notes had been double-coded (i.e. by M.V. and M.S.) to ensure that the work was done as accurately as possible. The record review was done many months after the interview had been completed, and the interview data were not available to the abstractor. The nurse also abstracted the pathological records (a) to confirm that the lesion was indeed malignant and (b) to discover whether or not there was histological evidence of axillary node involvement. Information recorded by the pathologist was not, of course, taken into account in the clinical staging.

A total of 747 women was interviewed. Of these 44 (5.9%) were excluded from the analysis because the cancer had been discovered by someone other than the woman herself. Staging information was obtained for 616 (87.1%) of the remaining 707 women. Our failure to obtain the necessary information for 91 women was due in part to missing case notes and in part to inadequate clinical information in some of the notes which were found. There were however, no important differences between the 616 women for whom staging was completed and the 91 women for whom it was not with regard to BSE history, age, oral contraceptive use or social class.

### Results

The relationship between the BSE history given by the

Correspondence: M.P. Vessey.
Received 25 June 1986; and in revised form, 13 October 1986.

**Table I** Clinical characteristics of breast tumour at presentation in relation to practice of breast self examination (BSE). (Data are percentages of women in each BSE group)

| | *History of breast self examination* | | | | |
|---|---|---|---|---|---|
| | | | *Done and taught* | | |
| | | | *Examination frequency* | | |
| *Tumour characteristics* | *Not done* | *Done but not taught* | *<Monthly* | *Monthly or more often* | *Total* |
| Size ≤2 cm | 33.2 | 34.4 | 43.6 | 45.3 | 37.5 |
| Skin of breast normal | 68.0 | 64.6 | 75.6 | 71.5 | 69.3 |
| Nipple normal | 86.7 | 89.6 | 86.6 | 88.9 | 87.7 |
| No deep attachment | 93.9 | 92.7 | 97.6 | 95.8 | 94.6 |
| $T_1$ | 27.2 | 26.0 | 40.2 | 41.7 | 32.1 |
| Stage 1 | 66.0 | 61.5 | 70.7 | 70.8 | 67.0 |
| Axillary nodes not palpable ($N_0$) | 74.1 | 71.8 | 79.3 | 80.6 | 76.0 |
| Number of women in BSE group | 294 | 96 | 82 | 144 | 616 |

The discrepancy in the results for $T_1$ tumours and for Stage 1 tumours reflects the fact that the effect of BSE is primarily on the $T_1/T_2$ ratio – i.e. the increase in $T_1$ tumours is compensated for by a reduction in $T_2$ tumours rather than in $T_3$ or $T_4$ tumours.

**Table II** Histopathological information on involvement of axillary nodes by disease in relation to breast self-examination

| | *History of breast self examination* | | | | |
|---|---|---|---|---|---|
| | | | *Done and taught* | | |
| | | | *Examination frequency* | | |
| *Axillary nodes* | *Not done* | *Done but not taught* | *<Monthly* | *Monthly or more often* | *Total* |
| (a) Histologically negative | 79 | 30 | 25 | 47 | 181 |
| (b) Histologically positive | 135 | 38 | 29 | 46 | 248 |
| (c) No mention of nodes in report | 80 | 28 | 28 | 51 | 187 |
| Total | 294 | 96 | 82 | 144 | 616 |
| % negative, nodal status known $\left\{\frac{100a}{a+b}\right\}$ | 36.9 | 44.1 | 46.3 | 50.5 | 42.2 |
| % with no report of positive nodes $\left\{\frac{100(a+c)}{a+b+c}\right\}$ | 54.1 | 60.4 | 64.6 | 68.1 | 59.7 |

patient and the clinical characteristics of the tumour at presentation (as abstracted from the case notes) is shown in Table I. Differences in tumour characteristics between those in the group not practising BSE and those in the practising but not taught group were small and inconsistent. Women who had been taught (and were practising) BSE, however, had a higher proportion of tumours with favourable characteristics than did women in the no BSE group. The difference was most marked in relation to tumour size. There was also a tendency for taught women examining their breasts monthly or more often to have slightly more favourable tumours than taught women examining their breasts less often than this.

Table II summarizes the available histopathological information on involvement of the axillary nodes by disease. Many pathologists' reports made no mention of the axillary nodes; in these circumstances it was impossible to tell whether no nodes had been submitted for examination or whether nodes had been submitted and had been found to be free of disease (we considered it very unlikely that the pathologist would fail to report positive nodes). However, no matter how the indeterminate category is handled, there is a

**Table III** Breast self examination in relation to age, oral contraceptive use and social class. (Data are percentages)

| | | *History of breast self examination* | | | | | |
|---|---|---|---|---|---|---|---|
| | | | | *Done and taught* | | | |
| | | | | *Examination frequency* | | | |
| | | *Not done* | *Done but not taught* | *<Monthly* | *Monthly or more often* | *Total* | *No. of women in group* |
| Age | -39 | 43.5 | 11.1 | 16.7 | 28.7 | 100.0 | 108 |
| | 40-49 | 47.1 | 14.8 | 12.7 | 25.4 | 100.0 | 244 |
| | 50-59 | 50.0 | 18.2 | 12.5 | 19.3 | 100.0 | 264 |
| Oral contraceptive use | Never | 50.6 | 17.8 | 11.7 | 19.9 | 100.0 | 377 |
| | Past | 42.8 | 12.2 | 16.2 | 28.8 | 100.0 | 198 |
| | Current[a] | 43.9 | 12.2 | 14.6 | 29.3 | 100.0 | 41 |
| Social class[b] (husband) | I | 37.3 | 11.9 | 15.3 | 35.5 | 100.0 | 59 |
| | II-IIINM | 46.4 | 16.7 | 16.3 | 20.6 | 100.0 | 209 |
| | IIIM-V | 47.3 | 16.2 | 10.4 | 26.1 | 100.0 | 241 |
| | Other | 57.0 | 14.0 | 13.1 | 15.9 | 100.0 | 107 |

[a]Use within year preceding diagnosis; [b]Registrar-General's classification. Questions were asked only about husband's occupation. The category 'other' includes no husband, students, armed forces.

**Table IV** Some tumour characteristics in relation to age, oral contraceptive use and social class. (Data are percentages of women in each group)

| | | *Tumour characteristics* | | | | | | | | |
|---|---|---|---|---|---|---|---|---|---|---|
| | | *Clinical* | | | | | | | *Pathological* | |
| | | *Size* $\leq 2$ *cm* | *Skin normal* | *Nipple normal* | *No deep attachment* | $T_1$ | *Stage* 1 | $N_0$ | *Axillary nodes negative, nodal status known* | *No report of positive axillary nodes* |
| Age | -39 | 44.2 | 83.3 | 95.4 | 95.4 | 43.5 | 64.8 | 72.2 | 43.1 | 69.4 |
| | 40-49 | 40.5 | 72.1 | 90.2 | 94.7 | 36.1 | 73.0 | 81.1 | 41.8 | 57.8 |
| | 50-59 | 31.8 | 61.0 | 82.0 | 93.2 | 23.9 | 62.5 | 72.7 | 42.8 | 57.6 |
| Oral contraceptive use | Never | 32.6 | 65.0 | 85.9 | 93.6 | 27.9 | 64.8 | 75.3 | 43.3 | 58.4 |
| | Past | 44.7 | 75.3 | 89.9 | 95.5 | 38.4 | 70.2 | 77.8 | 25.0 | 63.4 |
| | Current | 46.3 | 80.5 | 92.7 | 100.0 | 41.5 | 63.4 | 73.2 | 42.4 | 61.6 |
| Social class (husband) | I | 44.0 | 78.0 | 86.4 | 94.9 | 45.8 | 69.5 | 74.6 | 55.6 | 66.1 |
| | II-IIINM | 37.4 | 70.3 | 88.0 | 95.2 | 32.5 | 67.5 | 77.5 | 45.6 | 61.7 |
| | IIIM-V | 35.9 | 69.7 | 86.7 | 94.2 | 30.3 | 65.6 | 73.9 | 37.4 | 55.6 |
| | Other | 38.0 | 61.7 | 89.7 | 94.4 | 28.0 | 68.2 | 78.5 | 37.9 | 61.7 |

strong trend towards decreased axillary node involvement across the BSE groups. This trend is much more in evidence than is the trend concerning the clinical assessment of the axillary nodes shown in Table I.

We wondered whether the association between BSE history and tumour characteristics might be explained in terms of confounding by some other variable. Accordingly, we examined the effects of age, oral contraceptive use, social class and body weight. The last mentioned variable was found not to be a confounder but each of the other three variables was. Table III shows the relationship between BSE and age, oral contraceptive use and social class. As expected younger women, oral contraceptive users and women of high social class tended to have been taught BSE and to have been practising it more often than other women. In Table IV, the relationships between the same three confounding variables and tumour characteristics are examined. Younger women had tumours which were more favourable with regard to all the characteristics shown save for the percentage in Stage I and the percentage classed $N_0$. Much the same was true for oral contraceptive users. The pattern in relation to social class was more variable, but women in social class I were much less likely to have skin involvement than other women and had much more favourable axillary nodal findings on histological review (provided that only those of known nodal status were considered).

We used an additive multiple logistic model to adjust the data shown in Tables I and II for the effects of age, oral contraceptive use and social class. The results obtained are given in Table V. Although there is some reduction in the association between BSE and favourable clinical tumour characteristics, the data for size and for proportion of $T_1$ tumours remain encouraging and the trends are highly significant statistically. The figures also suggest a small advantage of BSE in relation to axillary nodal status assessed clinically. The data concerning the histopathological assessment of the axillary nodes continue to indicate a strong beneficial association with BSE.

### Discussion

The clinical findings reported here depend on the staging of tumours retrospectively from the data recorded in case notes, a procedure which is known to be inaccurate. In a previous study, however, using identical methods, we found case record based staging to provide a good indication of prognosis (see Greenberg *et al.*, 1985). Thus, in a series of

**Table V.** Breast tumour characteristics in relation to practice of BSE. Data are adjusted percentages in each group (taking into account age, oral contraceptive use and social class)

| | *History of breast self examination* | | | | | |
|---|---|---|---|---|---|---|
| | | | *Done and taught* | | | |
| | | | *Examination frequency* | | | |
| *Tumour characteristics* | *Not done* | *Done but not taught* | *<Monthly* | *Monthly or more often* | $\chi^2_{(3)}$ *hetero-geneity* | $\chi^2_{(1)}$ *linear trend* |
| Clinical | | | | | | |
| Size $\leq$2cm | 33.8 | 36.1 | 41.5 | 44.0 | 9.5[a] | 9.3[b] |
| Skin of breast normal | 68.6 | 66.0 | 74.2 | 68.9 | 3.3 | 0.3 |
| Nipple normal | 86.9 | 90.6 | 85.9 | 88.4 | 2.4 | 0.2 |
| No deep attachment | 94.3 | 93.4 | 97.6 | 96.0 | 4.9 | 2.3 |
| $T_1$ | 28.0 | 27.8 | 38.0 | 39.7 | 17.5[c] | 14.5[c] |
| Stage 1 | 66.0 | 62.1 | 70.2 | 70.4 | 4.1 | 2.2 |
| Axillary nodes not palpable ($N_0$) | 74.1 | 72.4 | 78.6 | 81.8 | 7.3 | 5.7[a] |
| *Pathological* | | | | | | |
| Axillary nodes negative, nodal status known | 37.2 | 44.6 | 45.4 | 50.2 | 10.6[a] | 10.1[b] |
| No report of positive axillary nodes | 54.5 | 61.7 | 64.0 | 67.8 | 16.6[c] | 16.3[c] |

[a]$P<0.05$; [b]$P<0.01$; [c]$P<0.001$.

Groupings used for age, oral contraceptive use and social class as shown in **Tables III and IV.** Percentages adjusted to overall distribution for all 616 women (or 429 women for the analysis dealing with those with axillary nodal status known).

654 women with breast cancer with long term follow up, the 10 year survival rate for the 162 with $T_1$ $N_0$ $M_0$ tumours was 72% and for the 212 with $T_2$ $N_0$ $M_0$ tumours was 59%. For the 23 with $T_1$ $N_1$ $M_0$ and the 94 with $T_2$ $N_1$ $M_0$ tumours, the survival rates were identical – both 46%. For the 163 remaining women, the survival rate was 32%. We thus consider our clinical data to be of prognostic value, but we recognise that their inaccuracy would also tend to obscure any association between BSE and early diagnosis. Accordingly, the true relationship between BSE and favourable clinical characteristics may well be greater than that demonstrated.

It can, of course, be argued that the association between BSE and favourable clinical tumour characteristics is not necessarily causal (i.e. that women who say they were taught and now practise BSE are for other reasons likely to present with more favourable tumours than other women), but the association remains after adjustment for identified confounding factors. Moreover, the case for a causal relationship is supported by the demonstration of an incremental effect of increasing degrees of implied effectiveness of BSE on the strength of the association. Having said this, it must be noted that the effect we have observed on clinical stage, although highly significant statistically, is nonetheless modest.

The results presented on the histopathological status of the axillary nodes are even more encouraging, although here too there must be some reservations about the adequacy of the data. The greater apparent effect on histologically assessed as against clinically assessed nodal status presumably reflects the inaccuracy of the clinical staging method. This histological data suggest that properly taught BSE could lead to a reduction of the order of 20–30% in the number of women presenting with positive axillary nodes at the time of diagnosis. It is plausible that an improvement in nodal status of this magnitude could be associated with an important increase in survival.

Our results are consistent with the five comparable studies reported since 1980 of which we are aware, bearing in mind that the definition of BSE and the non-BSE comparison group has been inconsistent. The proportions of tumours <2cm (or $\leq$2cm) in BSE and non-BSE groups in each study, defining the BSE group as women who practice BSE at least yearly (Huguley & Brown, 1981), several or more times yearly (Feldman *et al.*, 1981), ever (Foster & Constanza, 1984), at least three times a year (Smith *et al.*, 1980), and at least monthly (Senie *et al.*, 1981) were respectively 47% *vs.* 37%, 56% *vs.* 39%, 42% *vs.* 23%, 23% *vs.* 22% and 48% *vs.* 43%. In the last two studies the observed difference did not reach statistical significance. Two studies of different design from the United Kingdom, reporting the effect of a BSE booklet (Turner *et al.*, 1984) and BSE instruction (Philip *et al.*, 1984), also described a reduction in tumour size in the intervention group. The failure to show a significant reduction in tumour size by Smith *et al.* may be explained by the limited number of women (127) upon whom the analysis was based. The study by Senie *et al.* contained a high proportion (9%) of microscopic tumours.

All the studies mentioned also reported axillary node status based on pathological information. The degree of ascertainment of nodal status varied (and was not reported by Smith *et al.* and Feldman *et al.*). The proportions of women with positive axillary nodes in BSE and non-BSE groups (using the same definitions as before) were respectively 43% and 50% (Huguley & Brown, 1981), 46% and 61% (Feldman *et al.*, 1981), 39% and 56% (Foster & Constanza, 1984), 36% and 42% (Senie *et al.*, 1981), and 41% and 42% (Smith *et al.*, 1980). As before, the observed difference in the latter two studies was not statistically significant.

Our conclusion is that BSE, after adequate teaching, does lead to earlier diagnosis. The clinical importance of this lead time is hard to assess, but we are following up all the women in our study and will be able to report on survival in due course. There is no doubt that the specificity of BSE as a screening test is low and the costs in terms of false positive results, anxiety, suffering and use of medical resources may be high. The important question now is not whether BSE advances diagnosis, but whether the benefit is sufficient to outweigh the human and resource costs.

We would like to thank Moya Simmonds, Elizabeth Hilton and Judith Young for interviewing the women with breast cancer and the consultants at the participating hospitals for allowing us to include patients under their care. The Imperial Cancer Research Fund kindly provided financial support.

### References

FELDMAN, J.G., CARTER, A.C., NICASTRI, A.D. & HOSAT, S.T. (1981). Breast self examination, relationship to stage of breast cancer at diagnosis. *Cancer*, **47**, 2740.

FOSTER, R.S. & CONSTANZA, M.C. (1984). Breast self examination and breast cancer survival. *Cancer*, **53**, 999.

FOSTER, R.S., LANG, S.P., CONSTANZA, M.C., WORDEN, J.K., HAINES, C.R. & YATES, J.W. (1978). Breast self-examination practices and breast cancer stage. *N. Engl. J. Med.*, **299**, 265.

FRANK, J.W. & MAI, V. (1985). Breast self examination in young women: more harm than good? *Lancet*, **ii**, 654.

GREENBERG, E.R., VESSEY, M.P., McPHERSON, K., DOLL, R. & YEATES, D. (1985). Body size and survival in premenopausal breast cancer. *Br. J. Cancer*, **51**, 691.

GREENWALD, P., NASCA, P., LAWRENCE, C., HORTON, J., McGARRAH, R., GABRIELE, T. & CARLTON, K. (1978). Estimated effect of breast self examination and routine physician examinations on breast cancer mortality. *N. Engl. J. Med.*, **299**, 271.

HUGULEY, C.N. & BROWN, R.I. (1981). The value of breast self examination. *Cancer*, **47**, 989.

INTERNATIONAL UNION AGAINST CANCER (1968). TNM classification of malignant tumours. Geneva: UICC.

McPHERSON, K., NEIL, A., VESSEY, M.P. & DOLL, R. (1983). Oral contraceptives and breast cancer. *Lancet*, **ii**, 1414.

PHILIP, J., HARRIS, W.G., FLAHERTY, C., JOSLIN, C.A., RUSTAGE, J.H. & WIJESINGHE, D.P. (1984). Breast self examination: clinical results from a population based prospective study. *Br. J. Cancer*, **50**, 7.

SENIE, R.T., ROSEN, P.D., LESSER, M.L. & KINNE, D.W. (1981). Breast self examination and medical examination related to breast cancer stage. *Am. J. Publ. Hlth.*, **71**, 583.

SMITH, E.M., FRANCIS, A.M., POLISSAR, C. (1980). Effect of breast self examination practices and physician examinations on extent of disease at diagnosis. *Preventive Medicine*, **9**, 409.

TABAR, L., FAGERBERG, C., GAD, A., BALDETORP, L., HOLMBERG, L., GRONTOFT, O., LJUNGQUIST, U., LUNDSTROM, B., MANON, J., EKLUND, G., DAY, N. & PETTERSON, F. (1985). Reduction in mortality from breast cancer after mass screening with mammography. *Lancet*, **i**, 829.

TURNER, J., BLANEY, R., ROY, D., ODLING-SMEE, W., IRWIN, G. & MACKENZIE, G. (1984). Does a booklet on breast self-examination improve subsequent detection rates? *Lancet*, **ii**, 337.

VESSEY, M., BARON, J., DOLL, R., McPHERSON, K. & YEATES, D. (1983). Oral contraceptives and breast cancer: final report of an epidemiological study. *Br. J. Cancer*, **47**, 455.